# Development and characterization of a preclinical total marrow irradiation conditioning-based bone marrow transplant model for sickle cell disease

**DOI:** 10.3389/fonc.2022.969429

**Published:** 2022-09-06

**Authors:** Srideshikan Sargur Madabushi, Raghda Fouda, Hemendra Ghimire, Amr M. H. Abdelhamid, Ji Eun Lim, Paresh Vishwasrao, Stacy Kiven, Jamison Brooks, Darren Zuro, Joseph Rosenthal, Chandan Guha, Kalpna Gupta, Susanta K. Hui

**Affiliations:** ^1^ Department of Radiation Oncology, City of Hope National Medical Center, Duarte, CA, United States; ^2^ Department of Medicine, Division of Hematology/Oncology, University of California, Irvine, CA, United States; ^3^ Radiation Oncology Section, Department of Medicine and Surgery, Perugia University and General Hospital, Perugia, Italy; ^4^ Department of Clinical Oncology and Nuclear Medicine, Faculty of Medicine, Ain Shams University, Cairo, Egypt; ^5^ Department of Radiation Oncology, Mayo Clinic, Rochester, MN, United States; ^6^ Department of Radiation Oncology, University of Oklahoma Health Sciences Center (HSC), Oklahoma City, OK, United States; ^7^ Department of Pediatrics, City of Hope National Medical Center, Duarte, CA, United States; ^8^ Department of Radiation Oncology, Albert Einstein College of Medicine, Montefiore Medical Center, Bronx, NY, United States; ^9^ Department of Medicine, Division of Hematology, Oncology and Transplantation, University of Minnesota, Minneapolis, MN, United States; ^10^ Southern California Institute for Research and Education, Veterans Affairs (VA) Medical Center, Long Beach, CA, United States

**Keywords:** total marrow irradiation, bone marrow transplantation, sickle cell disease, engraftment, chimerism, mast cells, two-photon microscopy, histopathology

## Abstract

Sickle cell disease (SCD) is a serious global health problem, and currently, the only curative option is hematopoietic stem cell transplant (HCT). However, myeloablative total body irradiation (TBI)-based HCT is associated with high mortality/morbidity in SCD patients. Therefore, reduced-intensity (2–4 Gy) total body radiation (TBI) is currently used as a conditioning regimen resulting in mixed chimerism with the rescue of the SCD disease characteristic features. However, donor chimerism gradually reduces in a few years, resulting in a relapse of the SCD features, and organ toxicities remained the primary concern for long-term survivors. Targeted marrow irradiation (TMI) is a novel technique developed to deliver radiation to the desired target while sparing vital organs and is successfully used for HCT in refractory/relapsed patients with leukemia. However, it is unknown if TMI will be an effective treatment for a hematological disorder like SCD without adverse effects seen on TBI. Therefore, we examined preclinical feasibility to determine the tolerated dose escalation, its impact on donor engraftment, and reduction in organ damage using our recently developed TMI in the humanized homozygous Berkley SCD mouse model (SS). We show that dose-escalated TMI (8:2) (8 Gy to the bone marrow and 2 Gy to the rest of the body) is tolerated with reduced organ pathology compared with TBI (4:4)-treated mice. Furthermore, with increased SCD control (AA) mice (25 million) donor BM cells, TMI (8:2)-treated mice show successful long-term engraftment while engraftment failed in TBI (2:2)-treated mice. We further evaluated the benefit of dose-escalated TMI and donor cell engraftment in alleviating SCD features. The donor engraftment in SCD mice completely rescues SCD disease features including recovery in RBCs, hematocrit, platelets, and reduced reticulocytes. Moreover, two-photon microscopy imaging of skull BM of transplanted SCD mice shows reduced vessel density and leakiness compared to untreated control SCD mice, indicating vascular recovery post-BMT.

## Introduction

Sickle cell disease (SCD) is a global inherited red blood cell disorder that affects over 100 million people in the US and several million worldwide (1). The only curative treatment option currently available for this disorder is allogenic hematopoietic stem cell transplant (HCT) after myeloablative conditioning. Almost all of the currently available treatments are palliative, leaving SCD patients with poor quality of life due to extreme pain episodes, end-organ damage, and reduced life expectancy (2–4). Although the myeloablative-HCT regimen results in complete donor cell engraftment alleviating the disease features, long-term damage to organs is a major concern in patients with chronic state of SCD from palliative treatment. Therefore, an alternative standard of care is reduced-intensity conditioning and HCT, which results in reduced organ damage yielding transient intermittent/mixed chimerism, alleviating the disease features during that stage; however, it eventually results in graft failure and relapse (5, 6). Although partial chimerism leads to clinical improvement of SCD, pathological and hematological abnormalities, such as hemolysis, anemia, and vaso-occlusive crisis, are not recovered completely at low RBC chimerism (7). TBI-based dose escalation from 2 Gy to 4 Gy increased chimerism and reduced graft rejection in SCD patients (8). However, TBI-based increased dose also increases organ damage and the technique cannot be used to further improve chimerism and long-term engraftment.

Similarly, the myeloablative TBI regimen is commonly used for HCT in hematological malignancy for over five decades (9–13). However, there has been little or no long-term improvement in outcomes for patients with treatment-refractory acute leukemia. Previous efforts to further increase radiation using TBI could reduce the leukemia burden (14). However, toxicities related to TBI encountered during dose escalation offset any gains in overall survival (11). Therefore, there is an unmet need to develop targeted delivery of radiation and reduce radiation exposure to the organs to preserve organ function and improve the quality of life. To overcome this technological gap, Hui et al. first developed total marrow irradiation (TMI) using helical tomotherapy (15). We have successfully adopted the TMI technique escalating radiation dose to 20 Gy to the bone marrow (BM)-containing skeletal system while reducing the dose to other vital organs like liver, lung, and GI to enhance the anti-leukemic effect in young adults (≤55 years of age) and refractory/relapsed patients with leukemia; a Phase II (NCT02094794) trial shows a 2-year overall survival (OS) rate of 41% (16), in contrast to the <10% survival rate reported for similar patients (17).

Although the clinical development of TMI technology has led to many clinical trials worldwide, a lack of advanced preclinical technology has limited the scope of further scientific advancement. The conventional mouse TBI treatment lacks imaging to identify organs and a three-dimensional (3D) dosimetric model to calculate detailed organ dosimetry, and it ignores the dosimetric effect of tissue heterogeneity. We recently developed a novel multimodal image-guided preclinical TMI model for bone marrow transplant (BMT) using immunocompetent (C57BL/6) mice, which maintained long-term engraftment with reconstitution potential and reduced organ damage. However, the SS mouse model is highly radiosensitive, and previous studies have shown the myeloablative dose around 6 Gy, compared to 10–11 Gy, which is the standard in B6 mice. Furthermore, the pathology of SS mice organs is similar to human SCD with evidence of intravascular hemolysis, cardiomegaly, nephropathy, liver infarcts, vascular congestion, apoptotic Purkinje cells, pulmonary hemorrhages, and siderosis (excess iron deposit in heart, lungs, kidney, etc.) (18), which perhaps makes them more sensitive to radiation doses. Therefore, standard TMI doses used on B6 mice may still be toxic to SS mice. Hence, a feasibility study to test the tolerance of dose-escalated TMI in SS mice is essential.

We therefore successfully implemented our recently developed novel preclinical high-precision TMI to deliver a higher radiation dose to the BM while sparing vital organs (15, 19) in the Berkeley SS-BMT mouse model. Additionally, we have also recently successfully used TMI as an HCT conditioning regimen in three patients with SCD. However, post-HCT, it is unclear how TMI affects BM engraftment, hematological recovery, and organ toxicity. A recent study using our developed 3D image-guided TMI mouse BMT model suggests that for a successful engraftment with reduced organ damage, we need an optimal balance of radiation exposure to the BM and other vital organs (20). Therefore, in the current proof-of-concept study, we first evaluated the feasibility of dose escalation using TMI in SCD mice. After determining the tolerated TMI dose, next, we evaluated the effect of dose escalation on donor cell chimerism and recovery of SCD features in donor-engrafted SS mice. Dose escalation using TMI was tolerated by SS mice with reduced organ damage. For complete engraftment, dose escalation and increased donor cell number were essential, and furthermore, only long-term engrafted mice showed recovery of SCD phenotypes, suggesting a reversal of sickling features by healthy HbA RBCs post-TMI-BMT.

## Materials and methods

### Animals

All studies were performed in accordance with the Institutional Animal Care and Use Committee at City of Hope, National Medical Center, Duarte, CA. The C57B/L6 mice (stock #000664) were purchased from Jackson Laboratory, Maine, USA. The Berkeley SS mice (Hbatm1(HBA)Tow Hbbtm2(HBG1,HBB*)Tow [homozygous SS]) (Stock # # 003342) were purchased from Jackson Laboratory, Maine, USA and housed at the COH facility during the study. The donor Berkeley AA mice (*Hbatm1(HBA)Tow Hbbtm3(HBG1,HBB)Tow* [homozygous AA]) expressing normal human HbA were bred and maintained as previously described (21).

### Study design

The study aim is to determine whether dose-escalated TMI is feasible in an SCD mouse model. The primary end point is to assess the tolerated TMI dose in SCD mice and survival of mice at 30 days post-BMT. The SS mice will be treated with different doses of TMI and TBI and transplanted with donor AA mice BM cells, and survival will be assessed at day 30 post-BMT. The secondary end point of the study is to assess organ damage and measure subsequent short-term (7, 14, and 30 days post-BMT) and long-term donor cell chimerisms (3 months post-BMT). The respective organ toxicities (liver, kidney, spleen, femur, lung, and skin) at different time points will be assessed by histopathology. The details of the TMI treatment, BM transplantation, and histopathology analysis methods are given below in the respective methods and results section.

### TMI treatment plan

**Figure 1:**
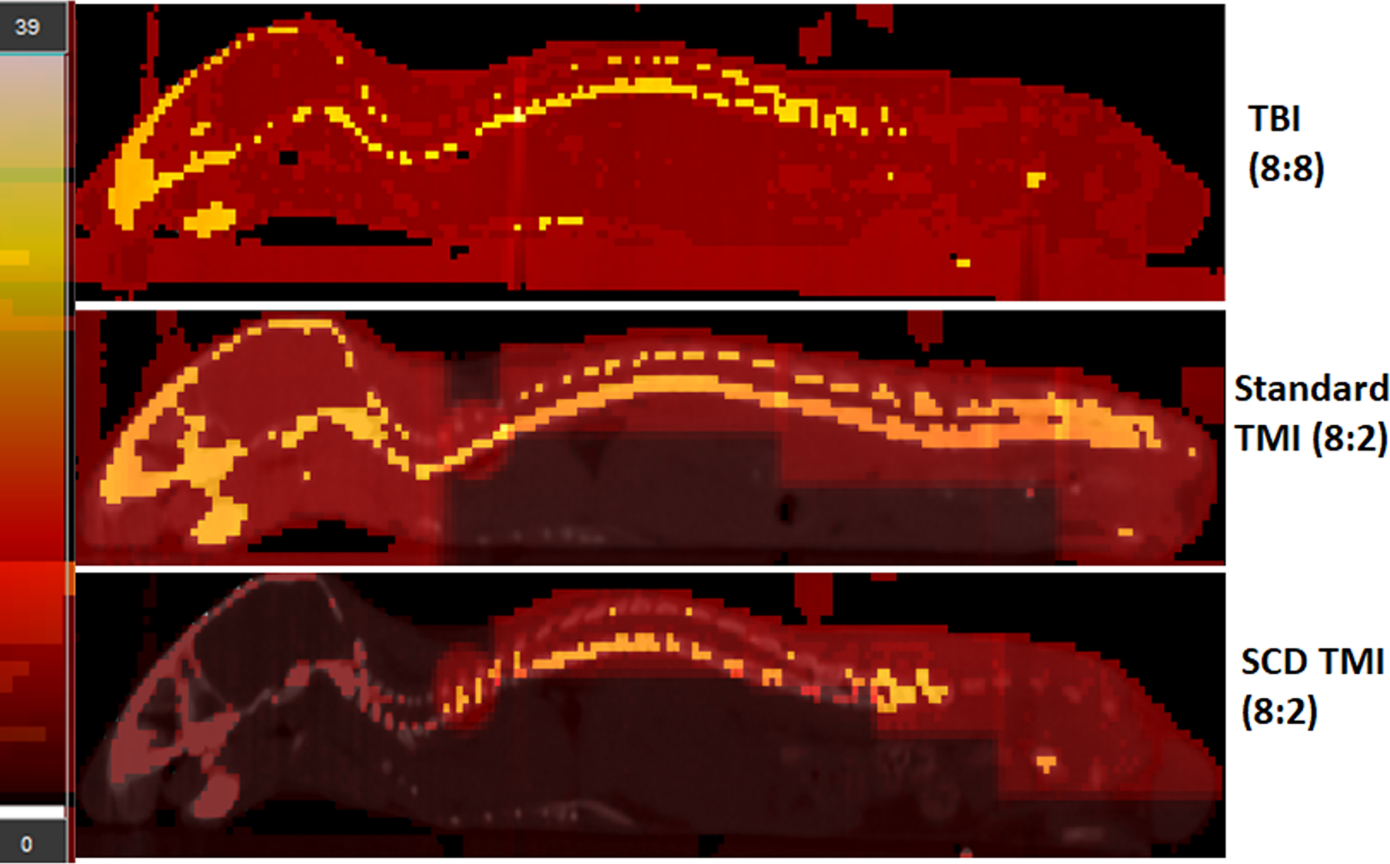
The modified TMI plan for SCD mouse. The TBI (8:8), standard TMI (8:2), and modified TMI (8:2) treatment plan for SCD are shown. The standard TMI (8:2) plan was modified by slightly adjusting the beam placements on the spine over the lung and kidney region. In addition, the dose to the skull was maintained at 2 Gy while the rest of the skeletal system received a prescription dose of 8 Gy. The dose painting clearly shows the difference in dose distribution in the new SCD TMI mice plan. The skull, face, and vital organs like liver, lung, and GI receive only ~2 Gy while the rest of the skeletal system is delivered with the prescribed dose. In addition, the TBI (8:8) mice dose painting clearly shows full prescription dose (~8Gy) to all the organs.

The TMI treatment was performed using the Precision X-RAD SMART Plus/225cx (Precision X-Ray, North Branford, CT, USA). The SS mice were treated with a TMI treatment plan according to a previous study but with some modifications (20). As the SS mice are radiosensitive and have enhanced organ damage due to sickling, particularly lung and kidney, we modified our TMI plan to further reduce doses to these organs. The isocenter of the T spine beam above the lung was moved up to further reduce doses to the lungs. For the kidneys, we divided the abdomen region into an upper and a lower section, and the isocenter for the beams for the abdomen region that has the kidneys was moved up, to further reduce the dose to the kidney. In a pilot study, we realized that a higher dose to the oral cavity was lethal to SS mice probably due to mucositis, and we kept the dose to the skull and oral cavity to a maximum of 2 Gy in the TMI plan. The comparison of the standard TMI plan and SCD modified TMI plan is shown in **Figure 1**. The radiation doses used in the study were as follows: TMI (4:0), TMI (4:2), TMI (6:2), TMI (8:2), TBI (2:2), and TBI (4:4). TMI (4:2) indicates a 4-Gy dose to the BM and a 2-Gy dose to the rest of the body. To keep the nomenclature consistent, for TBI 4 Gy, we use TBI (4:4), i.e., 4 Gy to the BM and 4 Gy to the rest of the body.

### Congenic bone marrow transplant study

For BMT studies, control HbAA-BERK (AA) mice were used as donors. AA mice are littermates of HbSS-BERK (SS) mice and hence have similar mixed genetic background. AA mice like SS mice do not have murine globin genes but exclusively express normal human hemoglobin A (human α and β globins). AA mice were bred as homozygous and the donor AA mice have CD45.1 immunophenotype. For the BMT study, host SS (CD45.2 or CD45.1/CD45.2 mixed immunophenotype) were treated with a single varying dose of TBI/TMI and 24 h later transplanted with different amounts of donor CD45.1 AA BM cells (5–25 million BM cells).

### Donor chimerism study

The peripheral blood was collected from the tail vein at different time points (7, 14, 30, and 90 days post-BMT) and donor chimerism was analyzed by flow cytometry using anti CD45.1 and CD45.2 antibody. The antibody-stained cells were acquired using BD fortessa and data were analyzed using FlowJo V10.1 software.

### Bone marrow cell harvest and stem cell staining

The BM cells were harvested and stained according to standard protocol (22). Briefly, the BM cells were collected by crushing the bones in PBS and filtered using a 30-micron MACS Smartstrainer (Cat # 130-098-458, Miltenyi Biotec, Auburn, CA). The RBCs were lysed from BM cells using ACK lysis buffer (Cat # A1049201 Gibco, Thermo Fisher Scientific, CA, USA) and single cells were stained with antibodies for CD45.1, CD45.2, lineage cells, cKit, Sca1, CD150, CD48, CD16/32, and CD34 (BioLegend, CA). The details about antibody are given in **Supplementary Materials**. The gating strategy for HSC was according to our previous published studies (22). The stained cells were acquired using a BD fortessa flow cytometer, and data were analyzed using FlowJo V 10.1.

### Hemoglobin electrophoresis

Hemoglobin electrophoresis is performed using a cystamine hemoglobin (Hb) cellulose acetate electrophoresis procedure (Adams et al., 2001). Briefly, 6 μl of whole blood is mixed with 9.5 μl of a cystamine solution containing 112 mg of cystamine dihydrochloride (Sigma Aldrich, Saint Louis, MO, USA), 0.9 ml sterile water, 0.5 ml of 0.3% ammonium hydroxide, and 100 μl of DTT at 15.43 mg/ml in a 1:3 dilution with sterile water. The mixture is incubated at room temperature for 15 min before applying to Titan III cellulose acetate plates (Helena Laboratories, Beaumont, TX), pre-soaked in SupraHeme buffer (Helena Laboratories) for 20 min. The loaded plates are then electrophoresed for 45 min at 280 V in SupraHeme buffer (Helena Laboratories). Gels are poststained using Ponceau S (Sigma), and washed in 5% acetic acid, methanol,and a Clear-Aid de-stain solution containing 300 ml of glacial acetic acid, 700 ml of methanol, and 40 ml of Clear-Aid (Helena Laboratories) for hemoglobin visualization. Interpretation: Using validated standards for human sickle (HbS) and normal human Hb (HbA), we can see a single band of HbS for homozygous pups (SS), a single band of human HbA for control pups (AA), and two bands comprising of one HbS and one HbA band for hemizygous (AS) pups.

### CBC and reticulocyte analysis

The peripheral blood was collected by cardiac puncture after euthanasia, in K3 EDTA tubes (BD biosciences). The CBC was analyzed using VetScan HM5 (Abaxis, Inc., Union City, CA, USA) (23). Reticulocytes were determined using BD Retic-Count (cat # 349204, BD Biosciences) according to the manufacturer’s instructions. Briefly, for 5 μl of blood, 1 ml of BD retic reagent was added, mixed and incubated in the dark for 30 min, and flow analyzed within 2 h of after incubation. As control, 5 μl of blood in PBS was used to normalize reticulocyte number.

### Multiphoton microscopy imaging

The MPM imaging using cranial window was carried out on SS mice according to a previous published study (24). Briefly, the day before microscopy imaging, a custom titanium head plate with an inner diameter of 8 mm was affixed directly on the frontal bone region of calvarium using Pearson PQ glass ionomer cement. To do headplate fixing, mice were first anesthetized using isoflurane, and surgery was performed to remove the skin and periosteum above the cranium. A stereotactic apparatus with a bite bar was utilized to keep the mouse stable during surgery. Herein, a headplate was used to stabilize the mouse head movement (even during breathing) while restraining the animal on the heated microscope stage. A 27-gauge catheter connected to an extension set is inserted into the mouse tail before loading mice on the microscope stage. The catheter allows the tail vein infusions of contrasts during imaging.

### Equipment setup and imaging

A Prairie ultima multiphoton microscope (Bruker Corporation, Billica, MA) with Olympus XLUMPlanFL 20× objective (1.00 NA water objective) was used for image acquisition of all images. Imaging is performed on the frontal bone region of the calvarium. During imaging, four-channel acquisition was kept at far red, red, green, and blue. The channel gains, wavelength, and the laser power change for time lapse (TSeries) images, vascular blood pool (ZSeries) images, and blood flow measurements, but we keep these parameters uniform for all mouse types.

### Image tiling

For tiling, images were acquired by collecting approximately a 2 × 3 grid series of overlapping z-stack images. The images were overlapping by 15% with a resolution of 512 by 512 pixels and a z-slice spacing of 15 µm. Tiled images were stitched together using ImageJ (Fiji) grid collection/stitching plug-in.

### Image analysis

In this study, we have analyzed the irradiation and BMT-induced alteration in vessel morphology and their physiology. Vascular diameter and the number of vessel branches per area were quantified using ImageJ (Fiji).

### Histopathology

After euthanasia, the tissues (liver, kidney, spleen, femur, lung, and skin) were harvested from respective mice and fixed in 10% Neutral Buffered Formalin (NBF) overnight and paraffin embedded according to SOP from the COH histology core. The formalin-fixed paraffin-embedded (FFPE) sections were stained with hematoxylin and eosin (H&E), Prussian Blue stain, and periodic acid Schiff–hematoxylin using standard protocols. Images were visualized using an Olympus microscope AX80 with a 10×, 20×, 40× eyepiece and an Olympus U-CMAD3 camera, with infinity analyze software from Lumenera Corporation. Morphologic findings of vascular congestion and iron deposits were analyzed according to the scoring system (18) that ranges from 0 for absent lesions up to a score of 6 for severe abundant lesions occupying 90%–100% of the field. For the kidney section, glomerular vascular congestion and renal tubular lesion were assessed as previously described (25). In H&E-stained kidney sections, a minimum of 30 glomeruli were evaluated/section under 400× magnification from 10 randomly selected fields of the renal cortex, glomerular vascular congestion was calculated as the percentage of total glomeruli with congestion present in at least 25% of the glomeruli, and results were averaged for each kidney. Tubular brush border thickness was assessed on periodic acid Schiff–hematoxylin-stained sections using a 0–4 grading scale: 0 for no changes; 1 for lesions involving <25% of the area; 2 for lesions involving 25%–50% of the area; 3 for lesions involving >50% of the area; and 4 for lesions involving nearly 100% of the area. Short-term damage assessment: TBI (4:4)-, TMI (8:2)-, and TMI (4:0)-treated mice 30 days post-BMT with age-matched untreated SS control (*n* = 3). Long-term damage assessment: TMI (8:2)-treated mice 90 days post-BMT (*n* = 3) and untreated age-matched SS control (*n* = 2) (25).

### Mast cell analysis in skin sections

Skin sections were stained with toluidine blue for mast cell analysis (23). Toluidine blue stain was prepared by dissolving 0.25 g of toluidine blue (Sigma-Aldrich) in 35 ml of distilled water, 15 ml of ethanol 100%, and 1 ml of HCl. After deparaffinization, the skin sections were incubated in toluidine blue for 1 min at room temperature, washed with distilled water, and air-dried. The stained specimens were observed under an Olympus microscope AX80 at (600× magnification) to count the MCs recognized by red-purple metachromatic staining color on a blue background. Mast cells were counted in 20 fields, 2 sections per slide, and expressed as total mast cell number, number of degranulated mast cells, and percentage of degranulated cells. Degranulated mast cells were defined as cells associated with ≥8 granules outside the cell membrane at 600× magnification as described previously (23).

### Statistical analysis

Statistical analyses were calculated using GraphPad Prism software (GraphPad Software Inc., La Jolla, CA, USA). Statistics were performed using the two-tailed unpaired *t*-test, and one-way ANOVA test, and the data are presented as mean ± SEM. When *p*-values were <0.05, the difference was considered significant. Ns = not significant, *p < 0.05, **p < 0.01, ***p < 0.001, ****p < 0.0001.

## Results

The SCD Berkeley SS mouse model was used in this study to evaluate the feasibility of dose-escalated TMI. In this study, we used our recently developed preclinical 3D-TMI model to develop a treatment plan for SS mice (**Figure 1**). However, the standard TMI plan was modified to further reduce the mean lung dose by ~18% and kidney dose by ~50% (**Table 1**). The SS mice due to the underlying pathophysiology of the disease are radiosensitive and therefore the dose to the skull and oral cavity was kept at 2 Gy for all TMI treatment.

**Table 1:** Comparison between the SCD TMI, Standard TMI (8:2), and TBI (8:8) plan.

	SCD TMI (8:2)	Standard TMI (8:2)	TBI (8:8)	SCD TMI (8:2) vs. Standard TMI (8:2)
Organs	Mean dose (Gy)	Mean dose (Gy)	Mean dose (Gy)	Difference %
Intestine	2.9	4.2	8.3	31
Liver	2.4	3.4	8	29.4
Lungs	4.5	5.5	9.3	18.2
Heart	2.2	2.9	8.3	24.1
Kidneys	3.7	7.4	8	50

The modified SCD plan reduces the mean doses on vital organs (liver: ~29%; lungs: ~18%; heart: ~24%; and kidney: ~50%) than the standard TMI (8:2) plan in B6 mice. TBI (8:8) delivers full prescription dose (~8 Gy) to all the organs.

**Figure 2:**
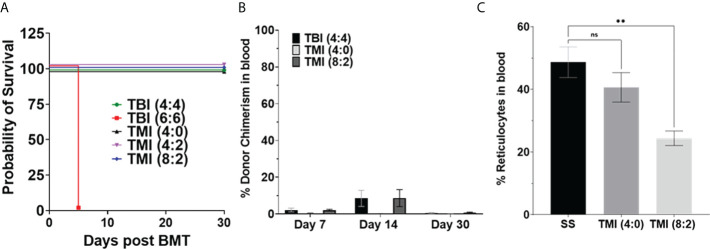
Sickle cell disease mice tolerated radiation dose and chimerism post-BMT. Berkeley SS mouse was treated with different dose of radiation and transplanted with 5 million donor AA bone marrow cells, and survival and chimerism were monitored over 30 days post-BMT. **(A)** Kaplan–Meier survival curve showing that TBI 6 Gy was lethal even after BMT, while TMI (8:2) was tolerated by SS mice (*n* ≥ 3). **(B)** Donor chimerism was measured in blood at D7, D14, and D30+ post-BMT. The data are representative of one experiment (*n* = 5) and was not repeated due to the limited availability of mice. **(C)** Peripheral blood reticulocytes were analyzed using BD Retic-Count by flow cytometry. The TMI (8:2) showed lower reticulocytes than TMI (4:0), perhaps due to initial donor chimerism seen at D14, suggesting that donor RBCs transplanted could temporarily rescue this phenotype. Significance was determined using two-tailed Student’s *t*-test and two-way ANOVA and was considered significant when *p*-value was <0.05. ns= non-significant, **p < 0.01.

First, we carried out a pilot study to evaluate the tolerated dose in the Berkeley SS mouse. The SS mouse was treated with TBI (4:4) and TBI (6:6) radiation and transplanted with 5 million donor whole BM cells from AA mouse to check the tolerated dose in SS mice. TBI 6 Gy was completely lethal while 4 Gy mice survived for 30 days post-BMT with no lethality (**Figure 2A**). We then evaluated the tolerated dose in the TMI model in SCD mice (*n* ≥ 3). Due to the limited availability of SS mice, we tested TMI (4:0) (4 Gy to BM, 0 Gy to the rest of the body) TMI (4:2) (4 Gy to BM, 2 Gy to the rest of the body), and TMI (8:2) (8 Gy to BM, 2 Gy to the rest of the body), increased the dose by 100%, and transplanted with donor BM cells. Because of the previous experience from the TMI mouse model and engraftment failure in TMI 12:0, we also chose to treat mice with a 2-Gy body dose while varying the dose to the BM.The SS mice tolerated TMI (4:0), TMI (4:2), and TMI (8:2) dose escalation.

Next, we evaluated the donor chimerism at D7, D14, and D30 post-BMT in peripheral blood. The SS mice were treated with TBI (4:4), TMI (4:0), and TMI (8:2) and transplanted with 5 million donor HbA BM cells. TBI (4:4) and TMI (8:2) had similar donor chimerism at D7 and D14 but higher than TMI (4:0)-treated mice; however, the engraftment failed (less than 10% chimerism) in all groups by day 30 (**Figure 2B**), suggesting a short-term engraftment failure. The initial chimersim observed on D7 and 14, maybe due to the mature cells from transplanted donor BM cells and this also resulted in reduced reticulocytes in TMI (8:2) treated mice (**Figure 2C**).

**Figure 3:**
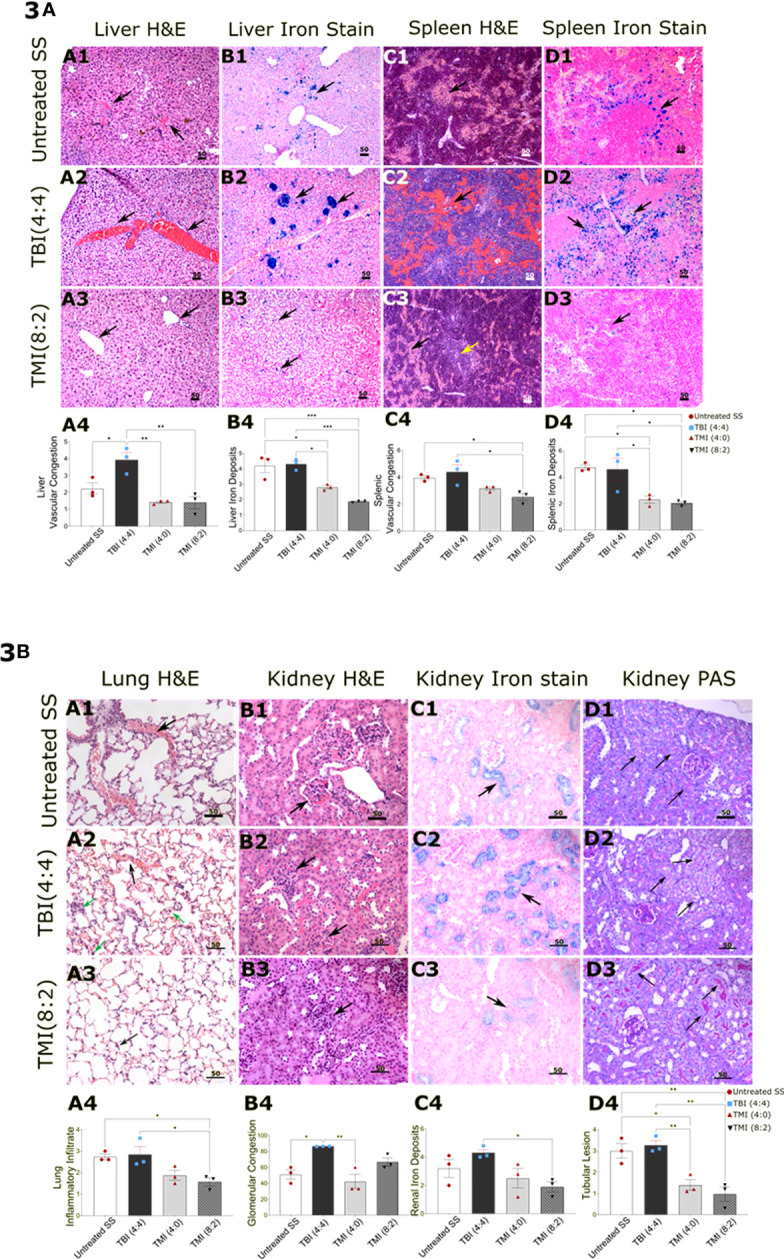
Comparison of histopathology in TBI, TMI, and untreated SS BMT control mice. Sections were stained with hematoxylin and eosin, Iron stain (Prussian Blue stain), and periodic acid Schiff–hematoxylin using standard protocols. Images were visualized using an Olympus microscope AX80 with a 10×, 20×, 40× eyepiece. Ten randomly selected fields, acquired from two sections per specimen from the liver, spleen, lungs, and kidneys, were scored for different parameters including vascular congestion, mononuclear inflammatory infiltrate, and iron deposits using Manci et al.’s scoring system (1). Glomerular vascular congestion was assessed on hematoxylin and eosin-stained sections and calculated as the percentage of total glomeruli with congestion present in at least 25% of the glomerulus as described (2). The microscopic findings in untreated SS are shown in figures **(A1–D1)**, TBI (4:4)-treated mice in **(A2–D2)**, and TMI (8:2)-treated mice in **(A3–D3)**, while **(A4–D4)** are showing the statistical analysis representative figures for the corresponding histopathological finding. **(A)** The histology sections demonstrate the hepatic findings showing more sinusoidal congestion in TBI (4:4) **(A2)**, black arrow) and siderosis [**(B2)**, black arrow] than TMI (8:2) **(A3, B3)**, black arrow). Spleen in TBI (4:4) showed more sinusoidal congestion **(C2)**, black arrow) and siderosis (**(D2)**, black arrow) than in TMI (8:2) [**(C3, D3)**, black arrow]. The yellow arrow in C3 represents preserved white pulp in TMI (8:2)-treated mice. **(B)** Pulmonary findings in the TBI (4:4) included more vascular congestion [**(A2)**, black arrow] and inflammatory infiltrate [**(A2)**, green arrow] than TMI (8:2) **(A3)**. Renal findings in TBI (4:4) included markedly congested glomerular (B2, black arrow) and denser iron deposits (**C2**, black arrow) than TMI (8:2) **(B3, C3)**. TBI (4:4) showed more renal tubular lesions marked by a significant loss in the tubular brush border lesion as observed in the analysis of PAS-stained slides [**(D2)**, black arrow]. Liver and spleen H&E and Iron stain original magnification, 100×. Lungs (H&E, Iron stain) and kidney (H&E, Iron stain, PAS) original magnification, 200×. Statistical analysis figures are generated by GraphPad software; ordinary one-way ANOVA, Tukey’s multiple comparisons test. Data are shown as mean ± SEM. *p < 0.05; **p < 0.01; ***p < 0.001. H&E, hematoxylin and eosin; PAS, Periodic acid Schiff; TBI, total body radiation; TMI, targeted marrow irradiation.

Although the chimerism failed in these mice, we wanted to evaluate the organ damage by radiation post-BMT in SS mice. Therefore, we evaluated the organ damage from TBI (4:4)-, TMI (4:0)-, and TMI (8:2)-treated mice and age-matched untreated SS mice (*n* = 3/group). We carried out histopathology, at 30 days post-BMT, on femur, lung, liver, kidney, and spleen (**Figures 3A, B**). We observed a statistically significant reduction in splenic sinusoidal congestion in TMI (8:2)-treated mice (*p*-value = 0.048 vs. untreated SS mice and *p*-value = 0.0126 vs. TBI-treated mice). This was associated with a significant reduction in splenic iron deposits compared to untreated SS in both TMI doses TMI (4:0) (*p*-value = 0.0248) and in TMI (8:2) (*p* = 0.0143) as well as a significant reduction in comparison to TBI (4:4)-treated mice [TBI (4:4) vs. TMI (4:0), *p* = 0.0328; TBI (4:4) vs. TMI (8:2), *p* = 0.0188]. The liver of TBI (4:4)-treated mice showed more sinusoidal congestion [*p* = 0.0283 vs. untreated SS, *p* = 0.0032 vs. TMI (4:0), and *p* = 0.0029 vs. TMI (8:2)]. TMI (4:0) and TMI (8:2) showed significantly reduced liver iron deposits compared to untreated SS mice (*p* = 0.0197 and = 0.0009, respectively); this significant reduction was also observed compared to TBI (4:4)-treated mice (*p* = 0.0136 and *p* = 0.0007, respectively). Lung tissue from TMI (8:2)-treated mice showed relatively 42% reduction in capillary congestion compared to TBI (4:4)-treated mice. TMI treated mice showed significant reduction in inflammatory infiltrate compared to untreated SS mice (*p* = 0.0454) and to TBI (4:4) (*p* = 0.0307), as well as significant reduction in lung iron store deposits in both TMI doses compared to TBI (4:4). Kidneys of TBI (4:4)-treated mice showed significantly congested glomerular capillaries compared to untreated (*p* = 0.0120) and TMI (4:0)-treated (*p* = 0.0032) mice. Both doses of TMI showed significantly less tubular brush border lesion as observed in the analysis of PAS-stained slides, in addition to reduced renal iron deposits in TMI (8:2)- compared to TBI (4:4)-treated (*p* = 0.0425) mice. Our observations from the morphological assessment of this pilot study support our hypothesis that TMI results in significantly less tissue damage compared to TBI. As TBI (4:4) caused more damage than TMI (8:2), which had ~2 Gy lower dose administered to these organs, for all further studies, we only considered treatment with 2 Gy or less body dose.

**Figure 4:**
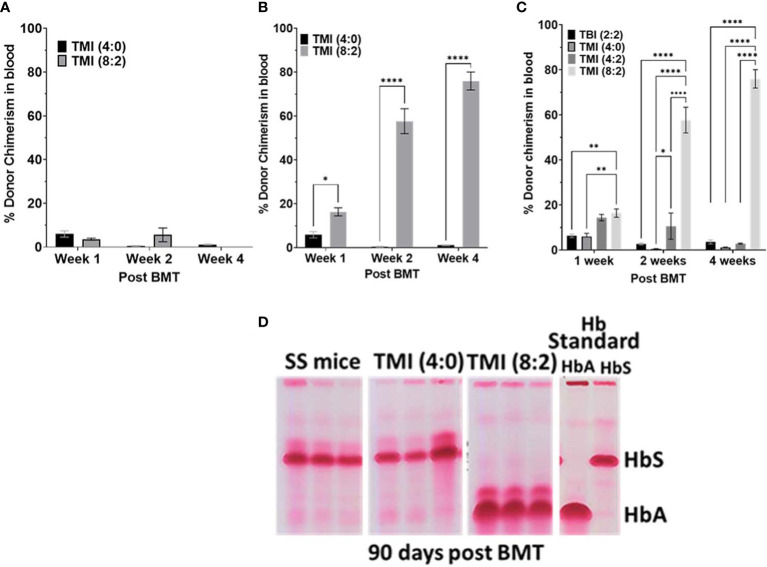
Effect of increasing donor cell number on chimerism and evaluation of blood hemoglobin for HbA and HbS. **(A, B)** The SS mice treated with TM (4:0) and TMI (8:2) transplanted with 10 million **(A)** and 25 million **(B)** AA donor BM cells and chimerism was measured in peripheral blood at D7, D14, and D30 post-BMT. **(C)** SS mice treated with different radiation doses were transplanted with 25 million AA donor BM cells and chimerism was checked at D7, D14, and D30 post-BMT in peripheral blood. Only TMI (8:2)-treated mice sustained chimerism at D30 post-BMT and was ~>65% (*n* ≥ 3). **(D)** CAE electrophoresis analysis of HbA and HbS 90 days post-BMT in peripheral blood. SS mouse was treated with TMI (4:0) and TMI (8:2) (Day −1) and 24 h later (Day 0) transplanted with 25 million donor AA BM cells. CAE analysis of TMI (8:2)- and TMI (4:0)-treated mice transplanted with 25 million cells on D90 post-BMT. Hb standard: from homozygous AA (HbA) and SS (HbS), and hemizygous AS (AFSC) mouse RBCs. Significance was determined using 2 way ANOVA and was considered significant when p value was < 0.05. * p<0.05, ** p< 0.01, **** p<0.0001.

Although SS mice tolerated dose-escalated TMI (8:2), they could not sustain engraftment with 5 million donor BM cells. Therefore, we evaluated whether SS mice could tolerate a high dose of radiation conditioning regimen for BMT with modifying donor cells and how this might impact optimization of engraftment/chimerism. This was achieved by transplanting 10 million and 25 million donor AA BM cells into TMI (4:0)- and TMI (8:2)-treated mice. Although 25 million donor cells increase the donor chimerism in both groups at D7, TMI (8:2) had the highest chimerism at all time points and showed sustained donor chimerism while TMI (4:0) failed at D30 post-BMT (**Figures 4A, B**). However, the TMI (8:2)-treated mice transplanted with 25 million donor BM cells showed a sustained long-term engraftment even at 90 days post-BMT (**Figure 4B**), which will be described later.

Furthermore, as 25 million cells showed sustained engraftment, we evaluated if TMI (8:2) dose escalation was essential for this sustained engraftment, or if a higher cell number was enough. We treated SS mice with TBI (2:2), TMI (4:2), and TMI (8:2) and transplanted 25 million HbA donor BM cells. At day 7 post-BMT, chimerism was much better in all groups, but TMI (4:2) and TMI (8:2) were higher than TMI (4:0) and TBI (2:2). However, only TMI (8:2)-treated mice could retain higher donor chimerism (>60%) by day 30 (**Figure 4C**), suggesting that both dose escalation and increasing donor cells were necessary for successful short- and long-term engraftment. We also measured HbA and HbS levels in peripheral blood post-BMT using CAE. With increasing donor BM cells (from 5 to 25 million), we observed an increase in HbA:HbS ratio by D14 in TBI (4:4) and TMI (8:2), while TMI (4:0) showed no change. However, 25 million donor BM cells showed the highest improvement in HbA:HbS ratio in TMI (8:2)-treated mice while TMI (4:0) still showed minimum to no change (**Figure 4D**), correlating with the enhanced donor chimerism in TMI (8:2) and failed chimerism in TMI (4:0).

**Figure 5:**
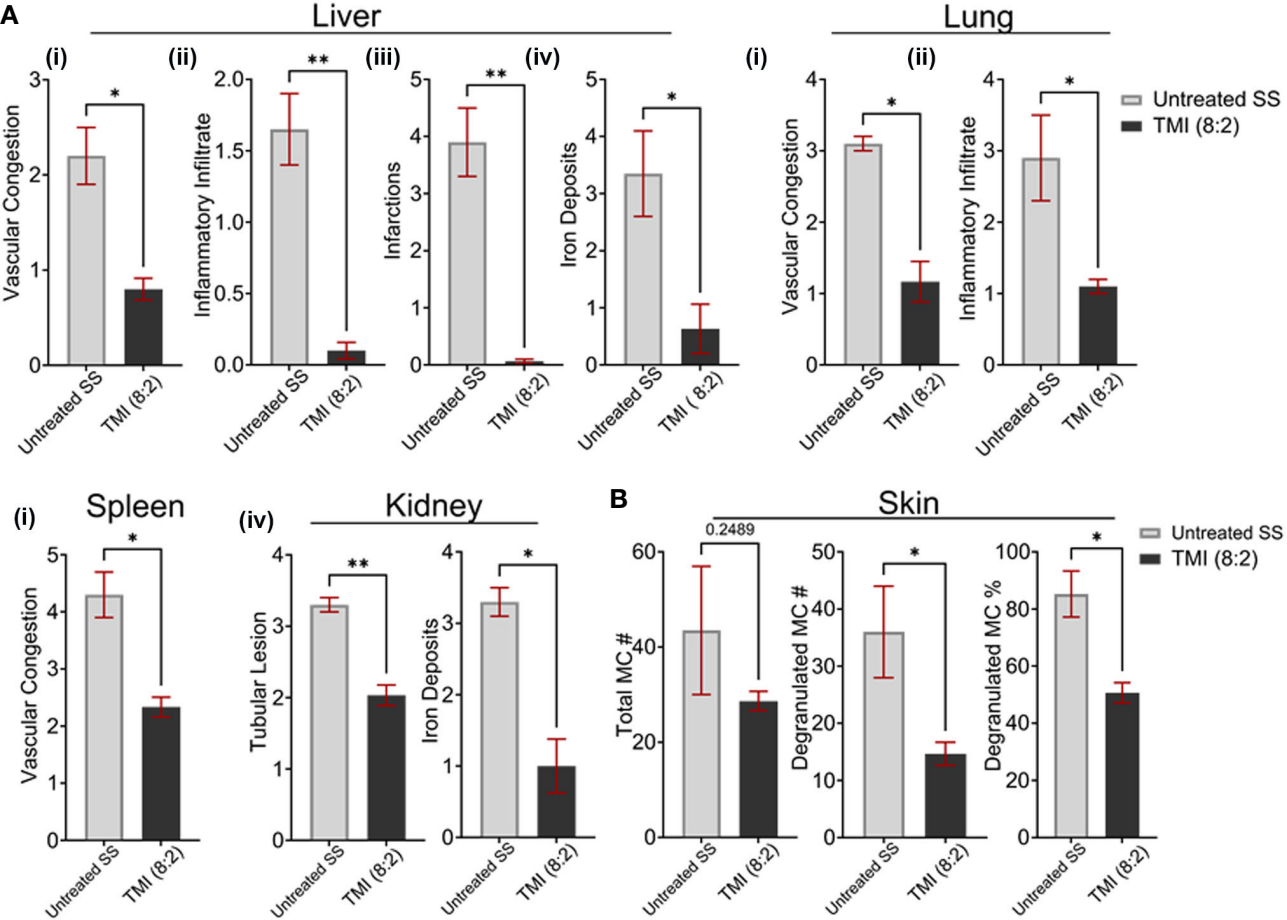
TMI (8:2)-treated mice long-term organ morphology assessment. We analyzed H&E- and PAS-stained sections of several organs for vascular congestion, inflammatory infiltrate infarcts, and iron deposits as described before (1). **(A)** Compared to untreated SS mice, the TMI (8:2)-treated mice showed significant reduction in (i) vascular congestion in lungs, liver, and spleen; (ii) inflammatory infiltrates in liver and lungs; (iii) infarction in liver infarction; and (iv) iron deposits in the liver and kidney. **(B)** Mast cell number, degranulated mast cell number, and percentage were determined from toluidine blue-stained skin sections from untreated SS mice and TMI (8:2)-treated mice 90 days post-BMT. The TMI-treated mice showed slightly lower but not significantly different mast cell numbers in the skin; however, the relative number and percentage of degranulated mast cells (activated) were significantly lower in TMI-treated mice than in untreated SS mice. Data are shown as mean ± SEM, analyzed with unpaired *t*-test, and two-tailed using GraphPad Prism *p < 0.05, *p < 0.01, **p < 0.01.

Since only TMI (8:2)-treated mice sustained donor chimerism long term, we compared the organ morphology of TMI (8:2)-treated mice with that of untreated SS mice to evaluate organ histopathology 90 days post-BMT. The respective organs’ FFPE sections were stained with H&E, PAS (kidney), and Prussian blue (iron deposits), and organ morphology was analyzed for vascular congestion, inflammatory infiltrates, infarcts, and iron deposits as described before (18, 25). Histopathological findings support that TMI (8:2) results in less tissue damage (**Figure 5A**) as evidenced by a significant reduction in vascular congestion (lung *p* = 0.0142, liver *p* = 0.0137, and spleen = 0.0135), inflammatory infiltrate (lung *p* = 0.0306, liver *p* = 0.0045), liver infarcts (*p* = 0.0034), and iron deposits in the liver (*p* = 0.0414) and kidney (*p* = 0.0205). At the level of renal tubules, untreated mice showed a significant loss in the tubular brush border when compared to TMI (8:2)-treated mice (*p* = 0.0082). BM in TMI (8:2) (cellularity ~70%) showed improvement in cellular differentiation and topography in comparison to the untreated (cellularity ~90%) mice that were packed with minimal fat spaces, thin trabeculae, and marked erythroid hyperplasia.

The mast cell activation has been shown to be responsible for enhanced pain, a sickle cell pathophysiology (23). We then investigated the total number of mast cells in skin of untreated SS mice and TMI (8:2)-treated SS mice 90 days post-BMT. The skin sections were stained with toulidine blue and the total number of mast cells and degranulating mast cells (activated) was calculated as described in *Materials and Methods*. The relative number of mast cells in TMI (8:2)-treated SS mice was slightly lower but not significantly different than age- and sex-matched untreated SS mice. However, the number (~45 vs. ~15) and percentage of degranulating mast cells (~85% vs. 50%) were signifcanlty lower than untreated SS mice (*p* < 0.05) (**Figure 5B**), suggesting that TMI (8:2) treatment and successful engraftment of donor cells may also reduce pain phenotype in SS mice.

**Figure 6:**
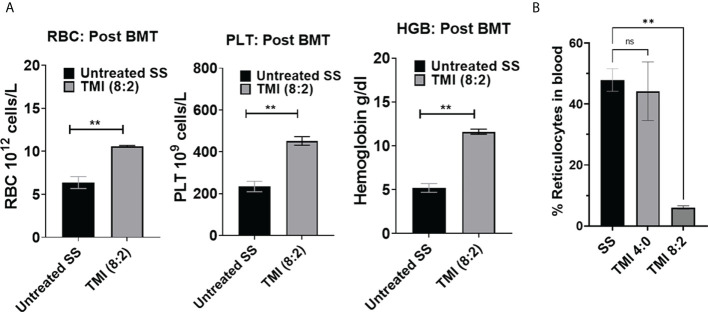
CBC analysis and peripheral blood reticulocytes from SS mice post-BMT. CBC was analyzed for TMI (8:2)-treated SS mice 90 days post-BMT. **(A)** RBC PLT and hemoglobin were significantly increased in BMT SS mice in comparison to untreated SS mice (*p* = 0.0042, 0.0067, and 0.0012, respectively), suggesting recovery of SCD phenotypes by BMT. **(B)** The reticulocytes were also significantly reduced in SS mice post-BMT (*p* = 0.0015) compared to untreated SS mice. Significance was determined using student's 't' test and 2 way ANOVA and was considered significant when p value was < 0.05. ns=non-significant, **p< 0.01.

The CBC analysis of peripheral blood at 3 months post-BMT from TMI (8:2)-treated mice showed a significant improvement in RBC numbers, Hb content, and platelets compared to the age- and sex-matched untreated SS mice (**Figure 6A**). The reticulocyte number in peripheral blood was also drastically reduced to ~5% in TMI (8:2)-treated mice compared to HbS control mice (~50%) (**Figure 6B**). These data suggest the recovery of sickle cell phenotype due to the replacement of sickle HbS RBCs with healthy HbA RBCs produced from AA donor cells.

**Figure 7:**
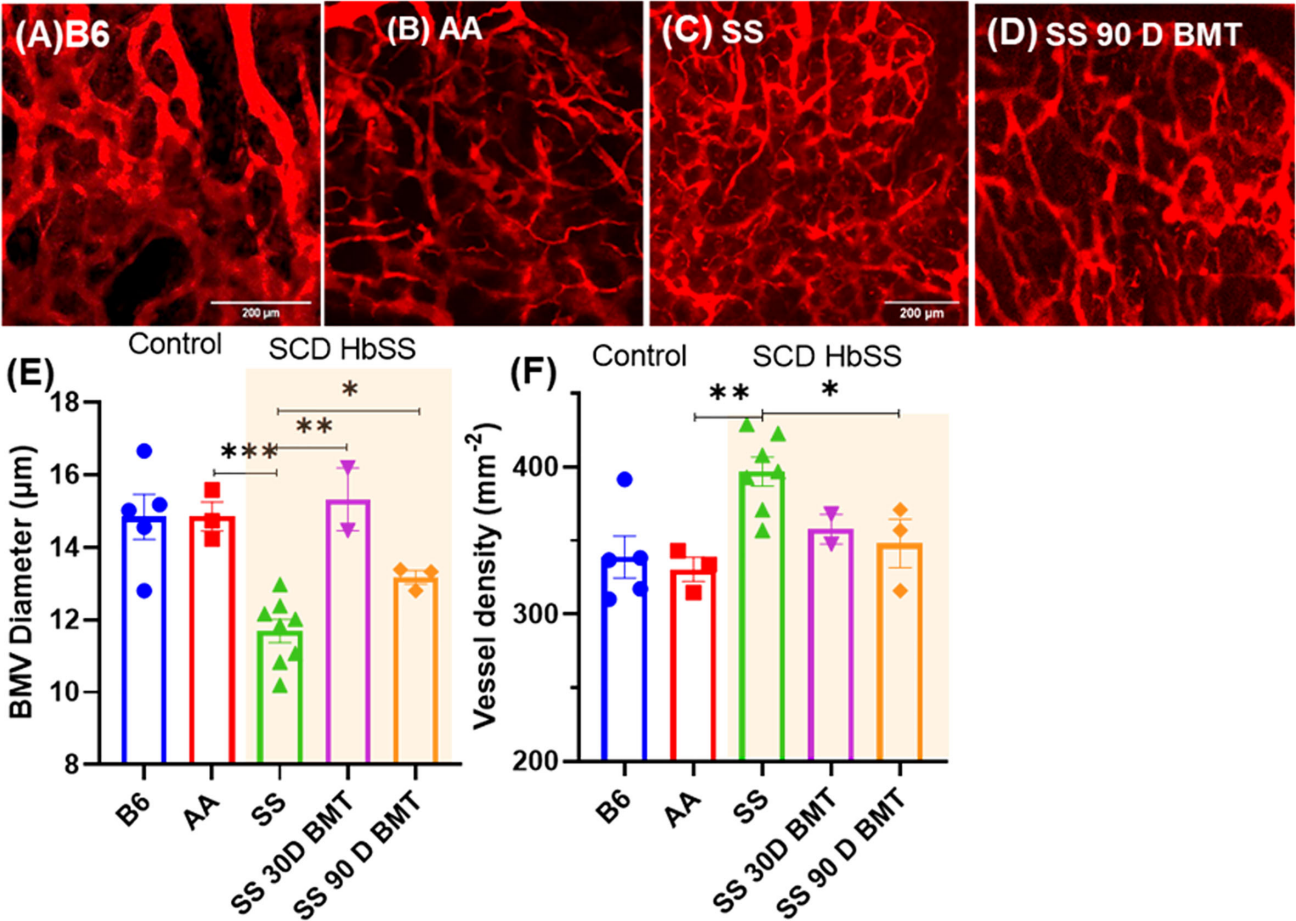
TPM imaging of microvasculature in SS mice and SS mice post-BMT. **(A–D)** TPM tiled images of SS mice and the controls, B6, AA, and 90 days post-BMT. The SCD mice have distinctly abnormal and disorganized BM vasculature **(C)** compared to that of control **(A, B)**; however, 90 days post-BMT in TMI (8:2)-treated mice, the microvasculature of the SS mice recovered and was like that of control mice **(D)**. **(E, F)** Vascular diameter and the number of vessel branches per area were quantified for the statistical measures. Statistically significant difference between average vessel diameter can be seen between SCD mouse and their controls **(E)** and the same is true for the vessel numbers/density **(F)**. Data shown as mean ± SEM for *n* ≥ 3 mice in each group. Significance determined using two-tailed unpaired *t*-test and considered significant when *p* < 0.05, *p<0.05, **p< 0.01, ***p<0.001.

Next, we investigated the microvascular structure of SCD mice post-TMI-BMT using our recently developed intravital multi photon microscopy (MPM) imaging using a cranial window. Using dextran as a blood pool agent, we visualized vessels and measured the average number of vessels and vascular diameter within the imaging window. The SCD mice showed a significanly higher number of vessels while vascular diameter was much smaller than control C57BL/6 and AA mice (**Figures 7A–F**). Interestingly, the TMI (8:2)-treated SCD mice 90 days post-BMT showed a microvascular structure similar to that of control AA mice, suggesting the recovery of microvascular niche post-BMT.

The hematopoietic stem and progenitor cells were analyzed in untreated control and TMI (8:2)-treated SS mice 3 months post-BMT. The Lin- cKit+ Sca1+ (LSK) HSCs were not significantly different than control HbS mice; however, we observed a slightly lower but not significantly different frequency of Lin- cKit+ Sca1- (LK) committed progenitors, particularly CMP and GMP in BMT mice. Interestingly, MEP progenitor cells that are committed to make megakaryocyte and erythroid cells were significantly increased in TMI (8:2)-treated mice post-BMT (**Supplementary Figure S1A**). This increase in MEP correlates with CBC data showing increased RBC number in peripheral blood in TMI-treated mice 90 days post-BMT. However, this is a limited mouse study and therefore more mouse data are required to confirm this observation.

## Discussion

This report is a first-in-field proof-of-concept study showing that the 3D image-guided TMI-based dose escalation is feasible for successful donor engraftment, rescue from SCD, and reduction of organ toxicity in an SCD mouse model. Due to the severity of the organ damage, TBI-based HCT is often avoided and palliative treatment is used until the disease becomes severe to accept. Reduced-intensity TBI was used as an alternative, which is less effective to cure SCD and toxicity remained high. Practically, there was no technological breakthrough to address this global health problem, and thus, curative option with good quality of life is severely limited for patients with SCD. Encouraged by our recent success of developing TMI delivering target-specific conformal radiation in HCT (15) and improving the clinical outcome of patients with high-risk leukemia (16), we conceptualized that TMI can be an effective treatment alternative for SCD patients. However, the lack of preclinical model limits our scientific understanding of the role of TMI in hematological disorder such as SCD and future clinical development.

SCD mice are radiosensitive, perhaps due to their underlying pathophysiology due to sickle RBCs. Like previous studies (26), the 6-Gy TBI dose was found to be highly toxic in a pilot study we conducted. Using a limited number of mice (*n* ≥ 3), we show that SCD mice could tolerate TMI (8:2) dose, that is, 8-Gy dose was delivered to the BM while 2 Gy was delivered to the rest of the body. Although no mice died in the pilot survival study, TMI (8:2) treatment sometimes resulted in death (~20%–40%, one to two out of five mice) in some studies. Death may be due to a combination of the pathophysiology of SCD mice and TMI treatment conditions, as the mice are anesthetized using isoflurane for over 30 min for TMI treatment. However, death of SS mice occurred around 4–6 days post-BMT, suggesting that cause of death is not due to hematopoietic failure, which usually occurs around 10–14 days post-BMT. Future studies are planned to understand the underlying cause of death by conducting necropsy of these mice.

After determining the tolerated TMI dose, we then evaluated the donor cell chimerism after dose escalation. The TBI (2:2)-, TMI (4:0)-, and TMI (4:2)-treated mice could not maintain chimerism by 30 days post-BMT even with increased 25 million donor cells, suggesting that these low-dose radiation was perhaps not sufficient to remove host HSC from their niche to create space for donor HSC to home and maintain a sustained long-term engraftment. Furthermore, in TMI (8:2) dose escalation, although no sustained engraftment was seen when 5 and 10 million donor cells were used, 25 million donor BM cells resulted in sustained engraftment, suggesting that increased dose could create space for donor HSC to home; however, sustained engraftment was possible only with higher donor BM cells. Similarly, in a previous study in SCD mice, increasing donor cell numbers from 10 to 50 million did not improve chimerism in nonmyeloablative Treosulfan conditioning; however, chimerism increased only when the conditioning dose of treosulfan was increased (27). Therefore, increased dose to the BM using TMI was also essential along with increased donor BM cells for sustained engraftment.

Although TBI (2:2) failed to engraft with 25 million donor cells, TBI (4:4) may have shown better chimerism and long-term engraftment with 25 million donor cells. However, organ damage in TBI (4:4)-treated mice and TMI (8:2)-treated mice clearly show that the 4-Gy dose to the organs damages more than 2 Gy used in TMI (8:2). Additionally, using TMI (8:4), we may be able to improve chimerism with lower donor cells, but the benefit of organ protection from TMI will be compromised. However, the use of TMI in combination with radiomitigators like thrombopoietin mimetics could be an alternative to protect organs by increasing the body dose to 4 Gy to reduce donor cell numbers. In addition, TPO is also known to stimulate HSC expansion post-BMT (28), perhaps improving donor chimerism by donor HSC expansion beyond radioprotection of organs. Therefore, further studies using such radiomitigators and reagents to expand HSC post-BMT should be tested in combination with TMI.

The stem cell analysis in long-term engrafted SCD mice showed an increased MEP progenitor population. These data could explain the recovery of RBC numbers and platelets in peripheral blood as MEP progenitors produce megakaryocytes (platelets) and erythroid cells (RBCs). The erythroid cell analysis using anti-CD71 and anti-Ter119 flow analysis from the spleen of TMI (8:2)-treated mice post-BMT also indicated normal erythropoiesis, unlike SCD mice that show accumulation of CD71 and Ter119 high immature cells (data not shown), consistent with increased percentage of reticulocytes in peripheral blood.

Long-term donor cell-engrafted mice also show recovery of reticulocyte percentage in peripheral blood, further suggesting recovery of erythropoiesis in SS mice post-BMT. The HbA:HbS ratio was also very high in long-term engrafted mice, further confirming the alleviation of HbS sickle cell phenotype. In addition, we also measured the activation of mast cells by determining the number and percentage of degranulated mast cells in the skin of SCD mice and post-BMT mice. The TMI (8:2) mice showed a reduced number and percentage of degranulated mast cells, suggesting the lower activation of mast cells. Mast cell activation has been shown to play an important role in pain perception in SCD (29, 30). Therefore, TMI (8:2) treatment not only improved chimerism but may have reduced pain in these mice. However, we need more studies to determine if donor engraftment or reduced skin dose in TMI (8:2) treatment or a combination of both was responsible for this phenotype.

The microvascular structure is altered significantly in SCD mice due to sickling of HbS RBC, which results in vaso-occlusion crisis. We have used cranial window and MPM to assess the BM vascular structure in the context of BMT in SS mice. The MPM imaging shows increased vascular density and reduced diameter in comparison to age- and sex-matched SS mice. Interestingly, the vessel density and diameter post-BMT was recovered to control AA mouse levels, indicating an association between recovery of microvascular structure and increased healthy HbA RBCs. Similarly, previous studies show that expression of HbF in SS mice resulted in normalization of microvascular and hemodynamic parameters by decreasing the sickling-induced transient vaso-occlusion events (31). However, in TMI (4:0) mice where chimerism completely failed, the vascular structure resembled untreated SS mice. This clearly suggests that donor engraftment and dose escalation enabled BM vasculature recovery. However, further studies are required to understand the mechanism of vascular niche remodeling in SCD mice post-BMT.

One of the limitations of the current study is that this is a congenic BMT study, resembling a matched donor-based allogeneic transplant in clinic. Matched donor is the first step to confirm the success of engraftment before moving towards mismatched or haploidentical donor settings. Haploidentical allogenic-HCT is often used for SCD patients due to the limited availability of HLA matched donors. We currently do not have AA mice in an allogenic mouse background. In the future, we can backcross the AA mice on an HLA mismatch genetic background mice like BALB/c and used the mismatched AA BM cells as donor to create an allogenic BMT study using TMI in SS mice.

Extramedullary hematopoiesis (EMH), a compensatory phenomenon to chronic hemolytic anemias, favors certain sites, mainly the spleen, and other sites such as the liver and the paraspinal regions of the thorax (32, 33). Thus, we have further evaluated EMH using histopathology. In SS untreated mice, we observed significant findings of EMH in the form of congested splenic sinusoids with hematopoietic cells including myeloid elements with different grades of maturation, erythroid cells, and megakaryocytes. These findings showed a reduction in the TMI (8:2) in treated mice after 90 days post-BMT, denoting that successful engraftment could reduce the EMH, one of the pathological features in SCD (data not shown). In agreement with this finding, there was a reduction of splenic EMH after hematopoietic stem cell transplantation in patients with myeloproliferative neoplasm-associated myelofibrosis (MPN-MF), another hematological disorder manifesting with splenomegaly secondary to extramedullary hematopoiesis (34).

In the future, we will use the currently developed TMI-SCD model to investigate how to increase donor chimerism and stabilize engraftment, leading to the long-term reversal of SCD phenotype. Our pilot data (not shown) and previous reports found that radiation exposure can increase hepatic thrombopoietin (35). TPO also plays a role in *HSC* self-renewal, proliferation, and differentiation (28, 36, 37). It is anticipated that reduced body (including liver) dose in TMI could reduce TPO, thus limiting the availability to support HSC expansion. Therefore, we will test whether varying radiation dose to liver could change TPO level (and other inflammatory cytokines), which could influence HSC homing and engraftment using TPO knockout mice. Finding a target (such as TPO) may allow us to use it along with TMI to improve engraftment (without increasing donor cells) and maintain normal BM hematopoiesis. Also, increasing BM-targeted radiation could adversely affect bone marrow environment (BME); therefore, future studies will require a thorough investigation on how to preserve BME.

In conclusion, we have evaluated the feasibility of dose-escalated TMI in the SCD mouse model. We show that dose escalation of TMI (8:2) is tolerated by SCD mice, but increased donor BM cells were essential for sustained engraftment. Higher donor chimerism reduces SCD features by increasing RBC number, platelets, hemoglobin content, and HbA:HbS ratio in RBCs while reducing reticulocyte number in peripheral blood. Therefore, this study provides the basis to conduct further studies using preclinical TMI to further improve the methodology and understand the mechanism for improved chimerism and reduction of SCD phenotypes. This preliminary study provides an opportunity to develop a new TMI-based preconditioning regimen for SCD patients that could reduce organ damage while increasing chimerism, and could therefore be a potential curative alternative to myeloablative HCT.

## Data availability statement

The original contributions presented in the study are included in the article/**Supplementary Material**. Further inquiries can be directed to the corresponding author.

## Ethics statement

This study was reviewed and approved by institutional animal care and use committee (IACUC). Protocol number #16064.

## Author contributions

SH and SSM: concept and experimental design, data analysis, interpretation of results, and manuscript writing and editing. JL and PV: BMT experimental design, data acquisition, and analysis. CG and JR: experimental design and manuscript editing. AMHA, DZ, and HG: experimental design and TMI treatment. HG and JB: MPM experimental design, data acquisition, analysis and interpretation of results, and manuscript editing. KG: experimental design, data analysis, and interpretation of histopathology and experiments with SCD and control mice and manuscript editing. RF: all histopathology analysis, interpretation, and co-wrote the manuscript. SK: phenotyping for sickle and normal human Hb, breeding and phenotyping control and sickle mice, and editing the manuscript. All authors contributed to the article and approved the submitted version.

## Funding

This work has been supported by NIH grants 2R01CA154491 (SH), RO1 HL147562 (KG), and U18 EB029354 (KG), and a Diversity Supplement 3R01HL147562-03S (SK). The content is solely the responsibility of the authors and does not necessarily represent the official views of the National Institutes of Health.

## Conflict of interest

KG: Honoraria: *Tautona Group*, *Novartis* and *CSL Behring*. Research Grants: *Cyclerion*, *1910 Genetics, Novartis*, Zilker LLC, *Grifols*, *UCI Foundation and SCIRE Foundation.* SH receives honoraria from and consults for Janssen Research and Development, LLC.

The remaining authors declare that the research was conducted in the absence of any commercial or financial relationships that could be construed as a potential conflict of interest.

## Publisher’s note

All claims expressed in this article are solely those of the authors and do not necessarily represent those of their affiliated organizations, or those of the publisher, the editors and the reviewers. Any product that may be evaluated in this article, or claim that may be made by its manufacturer, is not guaranteed or endorsed by the publisher.

## References

[1] Yawn BP Buchanan GR Afenyi-Annan AN Ballas SK Hassell KL James AH. Management of sickle cell disease: summary of the 2014 evidence-based report by expert panel members. Jama (2014) 312(10):1033–48. doi: 10.1001/jama.2014.10517 25203083

[2] Gladwin MT Vichinsky E. Pulmonary complications of sickle cell disease. New Engl J Med (2008) 359(21):2254–65. doi: 10.1056/NEJMra0804411 19020327

[3] Powars DR Chan LS Hiti A Ramicone E Johnson C. Outcome of sickle cell anemia: a 4-decade observational study of 1056 patients. Medicine (2005) 84(6):363–76. doi: 10.1097/01.md.0000189089.45003.52 16267411

[4] Ballas SK Lieff S Benjamin LJ Dampier CD Heeney MM Hoppe C. Definitions of the phenotypic manifestations of sickle cell disease. Am J hematol (2010) 85(1):6–13. doi: 10.1002/ajh.21550 PMC5046828 19902523

[5] Horan JT Liesveld JL Fenton P Blumberg N Walters MC. Hematopoietic stem cell transplantation for multiply transfused patients with sickle cell disease and thalassemia after low-dose total body irradiation, fludarabine, and rabbit anti-thymocyte globulin. Bone Marrow Transplantation (2005) 35(2):171–7. doi: 10.1038/sj.bmt.1704745 15531901

[6] Iannone R Casella JF Fuchs EJ Chen AR Jones RJ Woolfrey A. Results of minimally toxic nonmyeloablative transplantation in patients with sickle cell anemia and β-thalassemia. Biol Blood Marrow Transplantation (2003) 9(8):519–28. doi: 10.1016/S1083-8791(03)00192-7 12931121

[7] Iannone R Luznik L Engstrom LW Tennessee SL Askin FB Casella JF. Effects of mixed hematopoietic chimerism in a mouse model of bone marrow transplantation for sickle cell anemia. Blood (2001) 97(12):3960–5. doi: 10.1182/blood.v97.12.3960 11389040

[8] Bolanos-Meade J Cooke KR Gamper CJ Ali SA Ambinder RF Borrello IM. Effect of increased dose of total body irradiation on graft failure associated with HLA-haploidentical transplantation in patients with severe haemoglobinopathies: a prospective clinical trial. Lancet Haematol (2019) 6(4):e183–e93. doi: 10.1016/s2352-3026(19)30031-6 PMC6506220 30878319

[9] Thomas ED Clift RA Hersman J Sanders JE Stewart P Buckner CD. Marrow transplantation for acute nonlymphoblastic leukemic in first remission using fractionated or single-dose irradiation. Int J Radiat Oncol Biol Phys (1982) 8(5):817–21. doi: 10.1016/0360-3016(82)90083-9 7050046

[10] Brochstein JA Kernan NA Groshen S Cirrincione C Shank B Emanuel D. Allogeneic bone marrow transplantation after hyperfractionated total-body irradiation and cyclophosphamide in children with acute leukemia. New Engl J Med (1987) 317(26):1618–24. doi: 10.1056/nejm198712243172602 3317056

[11] Clift RA Buckner CD Appelbaum FR Bearman SI Petersen FB Fisher LD. Allogeneic marrow transplantation in patients with acute myeloid leukemia in first remission: a randomized trial of two irradiation regimens. Blood (1990) 76(9):1867–71. doi: 10.1182/blood.V76.9.1867.1867 2224134

[12] Petersen FB Deeg HJ Buckner CD Appelbaum FR Storb R Clift RA. Marrow transplantation following escalating doses of fractionated total body irradiation and cyclophosphamide–a phase I trial. Int J Radiat Oncol Biol Phys (1992) 23(5):1027–32. doi: 10.1016/0360-3016(92)90909-2 1639636

[13] Demirer T Petersen FB Appelbaum FR Barnett TA Sanders J Deeg HJ. Allogeneic marrow transplantation following cyclophosphamide and escalating doses of hyperfractionated total body irradiation in patients with advanced lymphoid malignancies: a phase I/II trial. Int J Radiat Oncol Biol Phys (1995) 32(4):1103–9. doi: 10.1016/0360-3016(95)00115-f 7607931

[14] Kal HB Loes van Kempen-Harteveld M Heijenbrok-Kal MH Struikmans H. Biologically effective dose in total-body irradiation and hematopoietic stem cell transplantation. Strahlenther Onkol (2006) 182(11):672–9. doi: 10.1007/s00066-006-1528-6 17072526

[15] Hui SK Kapatoes J Fowler J Henderson D Olivera G Manon RR. Feasibility study of helical tomotherapy for total body or total marrow irradiation. Med physics (2005) 32(10):3214–24. doi: 10.1118/1.2044428 16279075

[16] Stein A. Dose Escalation of Total Marrow and Lymphoid Irradiation in Advanced Acute Leukemia. Total Marrow Irradiation Eds. Wong J. Hui S. (2020). Springer, Cham. PP 69–75. doi: 10.1007/978-3-030-38692-4_4

[17] Duval M Klein J He W Cahn J Cairo M Camitta B. Hematopoietic stem-cell transplantation for acute leukemia in relapse or primary induction failure. J Clin Oncol (2010) 28(23):3730. doi: 10.1200/JCO.2010.28.8852 PMC2917308 20625136

[18] Manci EA Hillery CA Bodian CA Zhang ZG Lutty GA Coller BS. Pathology of Berkeley sickle cell mice: similarities and differences with human sickle cell disease. Blood (2006) 107(4):1651–8. doi: 10.1182/blood-2005-07-2839 PMC1895417 16166585

[19] Stein A Palmer J Tsai NC Al Malki MM Aldoss I Ali H. Phase I trial of total marrow and lymphoid irradiation transplantation conditioning in patients with Relapsed/Refractory acute leukemia. Biol Blood marrow Transplant J Am Soc Blood Marrow Transplantation (2017) 23(4):618–24. doi: 10.1016/j.bbmt.2017.01.067 PMC5382014 28087456

[20] Zuro D Madabushi SS Brooks J Chen BT Goud J Salhotra A. First multimodal, three-dimensional, image-guided total marrow irradiation model for preclinical bone marrow transplantation studies. Int J Radiat OncolBiolPhys (2021) 111(3):671–83. doi: 10.1016/j.ijrobp.2021.06.001 PMC8565655 34119592

[21] Sagi V Song-Naba WL Benson BA Joshi SS Gupta K. Mouse models of pain in sickle cell disease. Curr Protoc Neurosci (2018) 85(1):e54. doi: 10.1002/cpns.54 30265442

[22] Kumar B Madabushi SS. Identification and isolation of mice and human hematopoietic stem cells. In: Singh SR, rameshwar p, editors. somatic stem cells: Methods and protocols. New York, NY: Springer New York (2018). p. 55–68. 10.1007/978-1-4939-8697-2_4 30196401

[23] Vincent L Vang D Nguyen J Gupta M Luk K Ericson ME. Mast cell activation contributes to sickle cell pathobiology and pain in mice. Blood (2013) 122(11):1853–62. doi: 10.1182/blood-2013-04-498105 PMC3772495 23775718

[24] Brooks J Zuro D Song JY Madabushi SS Sanchez JF Guha C. Longitudinal preclinical imaging characterizes extracellular drug accumulation after radiation therapy in the healthy and leukemic bone marrow vascular microenvironment. Int J Radiat Oncol Biol Phys (2022) 112(4):951–63. doi: 10.1016/j.ijrobp.2021.10.146 PMC9038217 34767936

[25] Kasztan M Fox BM Speed JS De Miguel C Gohar EY Townes TM. Long-term endothelin-a receptor antagonism provides robust renal protection in humanized sickle cell disease mice. J Am Soc Nephrol (2017) 28(8):2443–58. doi: 10.1681/asn.2016070711 PMC5533228 28348063

[26] Pestina TI Hargrove PW Zhao H Mead PE Smeltzer MP Weiss MJ. Amelioration of murine sickle cell disease by nonablative conditioning and γ-globin gene-corrected bone marrow cells. Mol Ther Methods Clin Dev (2015) 2:15045. doi: 10.1038/mtm.2015.45 PMC4667717 26665131

[27] Devadasan D Sun CW Westin ER Wu LC Pawlik KM Townes TM. Bone marrow transplantation after nonmyeloablative treosulfan conditioning is curative in a murine model of sickle cell disease. Biol Blood marrow Transplant J Am Soc Blood Marrow Transplantation (2018) 24(8):1554–62. doi: 10.1016/j.bbmt.2018.04.011 29684562

[28] Fox N Priestley G Papayannopoulou T Kaushansky K. Thrombopoietin expands hematopoietic stem cells after transplantation. J Clin Invest (2002) 110(3):389–94. doi: 10.1172/jci15430 PMC151089 12163458

[29] Aich A Jones MK Gupta K. Pain and sickle cell disease. Curr Opin Hematol (2019) 26(3):131–8. doi: 10.1097/moh.0000000000000491 30893088

[30] Gupta K Jahagirdar O Gupta K. Targeting pain at its source in sickle cell disease. Am J Physiol Regul Integr Comp Physiol (2018) 315(1):R104–r12. doi: 10.1152/ajpregu.00021.2018 PMC6087885 29590553

[31] Kaul DK Liu XD Chang HY Nagel RL Fabry ME. Effect of fetal hemoglobin on microvascular regulation in sickle transgenic-knockout mice. J Clin Invest (2004) 114(8):1136–45. doi: 10.1172/jci21633 PMC522244 15489961

[32] Delicou S. Extramedullary Haemopoiesis in Hemoglobinopathies. J Hematol Transfus (2017) 5(2): 1066.

[33] Blouin MJ De Paepe ME Trudel M. Altered hematopoiesis in murine sickle cell disease. Blood (1999) 94(4):1451–9. doi: 10.1182/blood.V94.4.1451 10438733

[34] Pizzi M Gergis U Chaviano F Orazi A. The effects of hematopoietic stem cell transplant on splenic extramedullary hematopoiesis in patients with myeloproliferative neoplasm-associated myelofibrosis. Hematol Oncol Stem Cell Ther (2016) 9(3):96–104. doi: 10.1016/j.hemonc.2016.07.002 27521149

[35] Mouthon MA Vandamme M Gourmelon P Vainchenker W Wendling F. Preferential liver irradiation enhances hematopoiesis through a thrombopoietin-independent mechanism. Radiat Res (1999) 152(4):390–7. doi: 10.2307/3580223 10477915

[36] Kobayashi M Laver JH Kato T Miyazaki H Ogawa M. Thrombopoietin supports proliferation of human primitive hematopoietic cells in synergy with steel factor and/or interleukin-3. Blood (1996) 88(2):429–36. doi: 10.1182/blood.V88.2.429.bloodjournal882429 8695789

[37] Sitnicka E Lin N Priestley GV Fox N Broudy VC Wolf NS. The effect of thrombopoietin on the proliferation and differentiation of murine hematopoietic stem cells. Blood (1996) 87(12):4998–5005. doi: 10.1182/blood.V87.12.4998.bloodjournal87124998 8652812

