# Supplementary material for: Development and characterization of a preclinical total marrow irradiation conditioning-based bone marrow transplant model for sickle cell disease
Source: Front Oncol. 2022 Sep 6;12:969429. doi: 10.3389/fonc.2022.969429 (PMC9485604; doi:10.3389/fonc.2022.969429)
Supplement: Supplementary file 1 [file DataSheet_1.docx]

**Supplementary information**

**Flow cytometry antibody panel**

The Flow cytometry antibody panel used in the study.

| **Antibody** | **Target Population** | **Manufacturer** | **Cat#** |
| --- | --- | --- | --- |
| **Lineage cocktail** | (Lin neg) , HSPCs | Biolegend | 133310 |
| **Sca-1** | HSPCs, | Biolegend | 108134 |
| **c-kit** | HSPCs, MkP | Biolegend | 105811 |
| **CD16/CD32, FCR** | CMP, GMP, MEP | Biolegend | 305042 |
| **CD34** | LT-, ST-HSCs; | Biolegend | 152208 |
| **CD150** | LT-HSCs, MkP | Biolegend | 115941 |
| **CD48** | LT-HSCs | Biolegend | 103432 |
| **CD45.1** | Donor | Biolegend | 110728 |
| **CD45.2** | Host | Biolegend | 109814 |
| **Live dead** | live cells | Invitrogen | L34957 |

Lin: Lineage; HSPCs: Hematopoietic stem and progenitor Cells; MkP: Megakaryocyte progenitor; LT HSC: Long term Hematopoietic stem cells; ST HSCS: Short Term Hematopoietic Stem cells

**Supplementary figures**

**Supplementary Figure S1: Hematopoietic stem and progenitor cells (HSPCs) analysis in TMI (8:2) treated SS mice 12 weeks post BMT.** SS mice was treated with TMI (TMI 8:2) and transplanted with 25 million donor AA BM cells. The HSPCs were analyzed in BM 12 weeks post BMT. **A)** LK and LSK cells were not significantly different between untreated SS mice and BMT SS mice. **B)** Committed progenitors like CMP and GMP was not significantly different in BMT and untreated SS mice. **C)** However, the erythroid and megakaryocyte progenitors MEP was significantly increased post BMT in comparison to untreated SS mice (n=3, p=0.012), suggesting recovery of RBCs, as indicated by CBC data.
